# Two 27 MHz Simple Inductive Loops, as Hyperthermia Treatment Applicators: Theoretical Analysis and Development

**DOI:** 10.1155/2015/751035

**Published:** 2015-11-16

**Authors:** Vassilis Kouloulias, Irene Karanasiou, Maria Koutsoupidou, George Matsopoulos, John Kouvaris, Nikolaos Uzunoglu

**Affiliations:** ^1^2nd Department of Radiology, ATTIKON University Hospital, Medical School, University of Athens, Rimini 1, Haidari, 124 64 Athens, Greece; ^2^Microwave and Fiber Optics Laboratory, School of Electrical and Computer Engineering, Institute of Communication and Computer Systems, National Technical University of Athens, Heroon Polytechniou 9, 15780 Zografou, Greece; ^3^1st Department of Radiology, Aretaieion University Hospital, Medical School, University of Athens, Vassilissis Sofias 76, 115 28 Athens, Greece

## Abstract

*Background*. Deep heating is still the main subject for research in hyperthermia treatment. *Aim*. The purpose of this study was to develop and analyze a simple loop as a heating applicator. *Methods*. The performance of two 27 MHz inductive loop antennas as potential applicators in hyperthermia treatment was studied theoretically as well as experimentally in phantoms. Two inductive loop antennas with radii 7 cm and 9 cm were designed, simulated, and constructed. The theoretical analysis was performed by using Green's function and Bessel's function technique. Experiments were performed with phantoms radiated by the aforementioned loop antennas. *Results*. The specific absorption rate (SAR) distributions were estimated from the respective local phantom temperature measurements. Comparisons of the theoretical, simulation, and experimental studies showed satisfying agreement. The penetration depth was measured theoretically and experimentally in the range of 2–3.5 cm. *Conclusion*. The theoretical and experimental analysis showed that current loops are efficient in the case where the peripheral heating of spherical tumor formation located at 2–3.5 cm depth is required.

## 1. Introduction

Hyperthermia is a type of cancer treatment in which body tissue is exposed to higher than normal temperatures (up to 113°F = 45°C). The high temperatures inside the cells can damage and kill cancer cells or make them more likely to be affected by other forms of cancer therapy, such as radiation therapy and chemotherapy [1–6].

There are three main methods of hyperthermia: local, regional, and whole-body hyperthermia [1–8]. Local hyperthermia is used to heat a small area such as a tumor, using various types of energy to heat the tumor including microwave, radiofrequency, and ultrasound. Local hyperthermia aims to kill the cancer cells, coagulate the proteins, and destroy the blood vessels. Regional hyperthermia is used to heat a part of the body, such as a body cavity, organ, or limb, and it is usually combined with other types of cancer treatments. Finally, whole-body hyperthermia is used to treat metastatic cancer that has spread throughout the body, by raising the body temperature to 107-108°F = 41.6–42.3°C, using thermal chambers, hot water blankets, or warm-water immersion.

Theoretical, simulation, and experimental studies of hyperthermia are being carried out in order to provide us with an accurate temperature control procedure, while numerous clinical trials are being conducted for the effective combination of hyperthermia with radiation therapy and/or chemotherapy [2, 4–6].

Numerous applicators have been developed by various research groups in order to effectively perform hyperthermia treatment either at superficial or at deeper sites of the human body. Regarding the performance of resonant loop antennas, in some studies near-field wave impedance variations have been observed affecting the matching to the human body and the SAR [9–12].

Taking into account the abovementioned and with a view to effectively treating abdominal area superficial or deeper seated tumors we have studied the performance of loop antennas for potential use in hyperthermia clinical setups. More specifically, in this paper a 27 MHz inductive loop is examined as a potential tool for hyperthermia. Specifically, a theoretical electromagnetic analysis is carried out for the estimation of the electric field inside a semi-infinite space excited by circular current source. Numerical results for the SAR distribution are presented for two different current loops with radii 7 cm and 9 cm. Also, in order to validate the results, electromagnetic simulations of the two antennas radiating two human torso models were performed. The first model was simple, comprising a homogeneous cubic structure, and the second was a more detailed one, including a cylinder with layers of skin, fat, and muscle. Following that, for the experimental procedure, two inductive loop antennas were constructed with radii 7 and 9 cm, respectively. In addition, experiments were performed using human soft tissue phantoms at 27 MHz. Using the two antennas, the phantoms, and a multichannel electronic thermometer, temperature measurements were realized for a set of radiation sessions of various durations, depths, and distances with respect to the center of the circular loop.

## 2. Theoretical Electromagnetic Analysis

**Figure 1:**
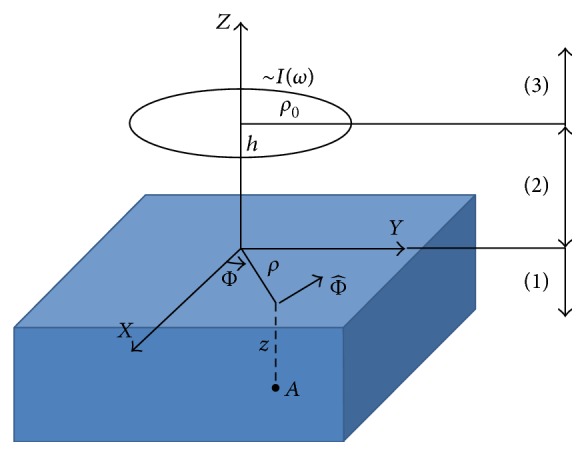
The geometry of the problem.

The purpose of the theoretical analysis is the estimation of the electric field at any arbitrary point inside a semi-infinite space with dielectric properties *σ*, *ε*_*r*_ *ε*_0_, and *μ*_0_, excited by a circular current source. The geometry of the problem is depicted in Figure 1, where *h* is the distance between the antenna and the dielectric, *ρ*_0_ is the radius of the antenna, *I*(*ω*) is the current with angular frequency *ω*, *A* is the point of interest in depth *z* inside the dielectric, and $\rho =\sqrt{x^{2}+y^{2}}$.

In order to solve this problem, Green's function technique is adopted. By virtue of the cylindrical symmetry of the problem, cylindrical coordinates are used.

The following expressions describe electric-type Green's function in the three regions of space. Region 1 demonstrates the area inside the dielectric media, Region 2 demonstrates the area between the antenna and the dielectric, and finally Region 3 demonstrates the area above the antenna.

*Region 1*. Consider

(1) $$\begin{array}{c} E_{1}\left(\;\rho ,z\;\right)=\overset{+\infty }{\underset{-\infty }{\int }}d\lambda J_{1}\left(\;\lambda \rho \;\right)A\left(\;\lambda \;\right)e^{-\mu_{\alpha }z}. \end{array}$$

*Region 2*. Consider

(2) $$\begin{array}{c} E_{1}\left(\;\rho ,z\;\right)=\overset{+\infty }{\underset{-\infty }{\int }}d\lambda J_{1}\left(\;\lambda \rho \;\right)\left[\;B\left(\;\lambda \;\right)e^{-\mu_{\alpha }z}+C\left(\;\lambda \;\right)e^{\mu_{\alpha }z}\;\right]. \end{array}$$

*Region 3*. Consider

(3) $$\begin{array}{c} E_{3}\left(\;\rho ,z\;\right)=\overset{+\infty }{\underset{-\infty }{\int }}d\lambda J_{1}\left(\;\lambda \rho \;\right)D\left(\;\lambda \;\right)e^{-\mu_{\alpha }z}, \end{array}$$

where *A*(*λ*), *B*(*λ*), *C*(*λ*), and *D*(*λ*) are unknown coefficients to be determined, *J*_1_(*λρ*) is the Bessel function of the first kind, and $\mu_{\alpha }=\sqrt{\lambda^{2}-k_{0}^{2}}.$

Hereupon, the unknown coefficients of the integral of cylindrical waves are determined by the boundary conditions on the interfaces *Z* = 0, *h*.

In order to satisfy the continuity of the electric field, the boundary condition on the interface *Z* = 0 between Regions 1 and 2 is imposed:

(4) ∫−∞+∞dλJ1λρAλ=∫−∞+∞dλJ1λρBλ+Cλ.

And the following equation can be obtained:

(5) $$\begin{array}{c} A\left(\;\lambda \;\right)=B\left(\;\lambda \;\right)+C\left(\;\lambda \;\right). \end{array}$$

Between Regions 1 and 2 magnetic fields *H*_1_ and *H*_2_ are generated. For unit vector $\hat{n}$ which is vertical in the interface we have $\hat{n}\times \left(H_{1}-H_{2}\right)=J$, where *J* is the surface current density. On the interface between Regions 1 and 2 there is no current density so $\hat{n}\times \left(H_{1}-H_{2}\right)=0$.

In order to satisfy the continuity of the magnetic field, the boundary condition on the interface *Z* = 0 is then imposed by implementing the following expression:

(6) $$\begin{array}{c} \frac{\partial E_{1}}{\partial z}=\frac{\partial E_{2}}{\partial z}. \end{array}$$

We have

(7) $$\begin{array}{c} A\left(\;\lambda \;\right)\mu_{i}=B\left(\;\lambda \;\right)\mu_{\alpha }+C\left(\;\lambda \;\right)\mu_{\alpha }. \end{array}$$

Following that the boundary conditions on the interface *Z* = *h* between Regions 2 and 3 are imposed. In order to satisfy the continuity of the electric field the following equation is obtained:

(8) ∫−∞+∞dλJ1λρBλe−μαh+Cλeμαh=∫−∞+∞dλJ1λρDλe−μαh,

(9) Bλe−μαh+Cλeμαh=Dλe−μαh.

Finally, due to the continuity on the interface between Regions 2 and 3 we have

(10) $$\begin{array}{c} \hat{n}\times \left(\;H_{2}-H_{3}\;\right)=J, \end{array}$$

where *J* is the current density due to the loop which is considered to be placed on the interface of the aforementioned regions. Consider $\hat{n}=\hat{z}$ so

(11) z^×H2=jωμ0φ^∂E2∂z,z^×H3=jωμ0φ^∂E3∂z.

For the current density it applies

(12) $$\begin{array}{c} J=\delta \left(\;\rho -\rho_{0}\;\right)I_{0}\hat{\varphi }, \end{array}$$

where *I*_0_ is the current which is flowing through the loop and *δ*(*ρ* − *ρ*_0_) is the Dirac delta function.

Using (10), (11), and (12) and for *Z* = *h*, the following equation can be obtained:

(13) $$\begin{array}{c} {\left.\;\frac{\partial E_{3}}{\partial z}-\frac{\partial E_{2}}{\partial z}\;\right|}_{z=h}=j\omega \mu_{0}I_{0}\delta \left(\;\rho -\rho_{0}\;\right). \end{array}$$

But it applies

(14) $$\begin{array}{c} \delta \left(\;\rho -\rho_{0}\;\right)=\frac{1}{2\pi }\overset{+\infty }{\underset{-\infty }{\int }}e^{jk\left(\rho -\rho_{0}\right)}dx\;. \end{array}$$

Based on the above expression and by implementing Jacobi's theorem and Fourier transforms the following equation can be obtained:

(15) $$\begin{array}{c} \frac{\delta \left(\rho -\rho_{0}\right)}{\rho }=\overset{\infty }{\underset{0}{\int }}\lambda d\lambda J_{1}\left(\;\lambda \rho \;\right)J_{1}\left(\;\lambda \rho_{0}\;\right). \end{array}$$

And (13) becomes

(16) ∫−∞∞−μαDλJ1λρe−μαh−−μαBλe−μαh+μαCλe−μαhJ1λρdλ=−jωμ0l0ρ0∫0∞λ dλJ1λρJ1λρ0.

*ρ* has been replaced with *ρ*_0_ in the above equation because the limit lim_*ε*→0_⁡[*δ*(*ρ* − *ρ*_0_)] converges for *ρ* → *ρ*_0_, and (16) becomes

(17) −μαDλeμαh+μαBλe−μαh−μαCλe−μαh=−jωμ0I0ρ0λJ1λρ0.

Consequently, the four unknown coefficients *A*(*λ*), *B*(*λ*), *C*(*λ*), and *D*(*λ*) can be derived from four linear equations (5), (7), (9), and (17) with very low computational cost. So it is evident that the electric field can be calculated inside and outside the dielectric material, at any point of the described configuration.

## 3. Materials and Methods

### 3.1. Simulation Setup

**Figure 2:**
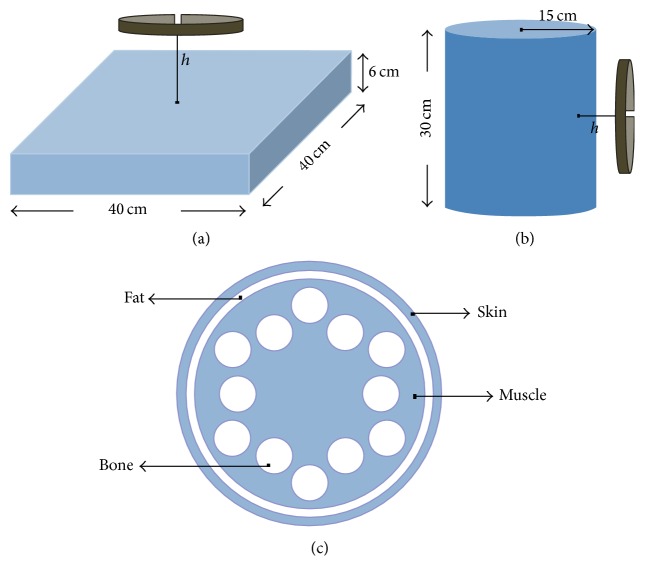
Geometrical configurations of (a) the cubic structure and (b) the cylindrical structure for the loop antenna simulation setups. (c) Schematic of the different layers of body tissues inside the cylindrical structure.

The circular current source was modeled by two inductive loop antennas with 2 cm height, 1 mm thickness, and radii 7 cm and 9 cm, respectively. The antennas were used as radiators placed 1 cm above two different setups:

(a) A homogeneous cubic structure with 40 cm × 40 cm × 6 cm dimensions (Figure 2(a)): the material used for the simulation has *ε*_*r*_ = 113 and *σ* = 0.62 Si/m representing soft tissues [13].
(b) A cylinder of 30 cm height and 15 cm radius (Figure 2(b)): the structure consisted of a cylinder of muscle tissue with 13 cm radius and it is covered by two layers of fat and skin, respectively, with 1 cm thickness each (Figure 2(c)). Smaller tubes of 2 cm radius lie inside the internal cylinder representing bone tissues. The dielectric properties of the body tissues used for the second setup are shown in Table 2 [14].

## 4. Experimental Setup

### 4.1. Radiator Development

Two inductive loop antennas were designed and constructed with radii 7 and 9 cm, respectively. The antenna dimensions have been chosen to meet the following criteria:

- Dimensions of the area for hyperthermia treatment.
- Satisfactory operation of loops at 27 MHz.
- Satisfactory penetration depth and spatial resolution.

**Figure 3:**
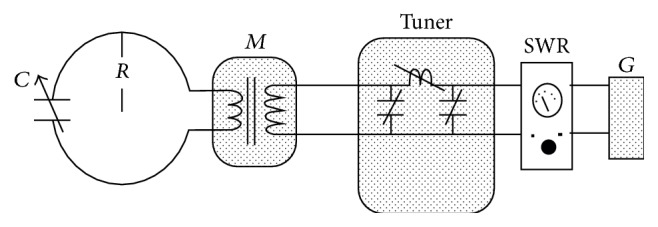
Experimental setup.

The radiators consist of three parts (Figure 3):

(a) The main radiator which consists of a brass loop with 2 cm height and 1 mm thickness.
(b) The balanced-unbalanced transformer (*M*) 16 : 4 with the signal input.
(c) The resonance capacitor (*C*).

The antennas receive power by a generator (*G*) 27.12 MHz and 100 WRMS. A measuring bridge for standing waves (SWR) is inserted in the circuit. The resonance is achieved by the use of a tuner which consists of variable capacitors and coils as shown in Figure 3.

The resonance criteria were

- the minimization of standing waves by appropriate handling of the system's variable inductions and capacities,
- increase of the standing waves when the antenna was removed from the phantom's dielectric material.

### 4.2. Phantom Construction

**Figure 4:**
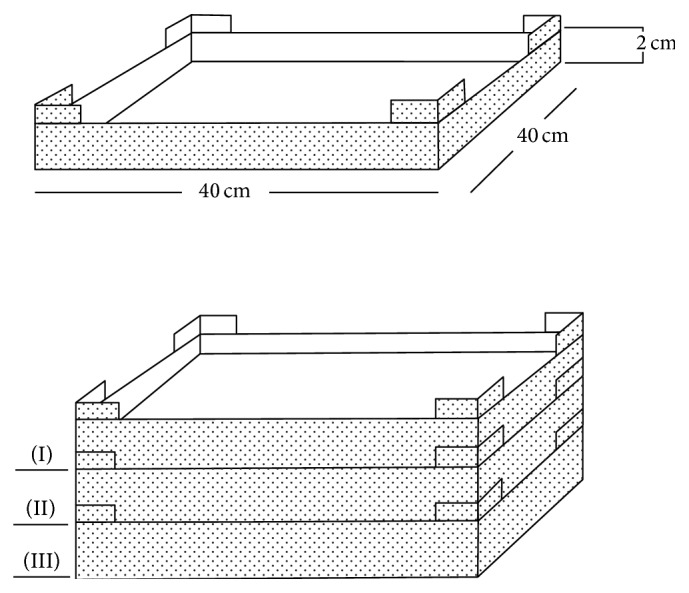
Plexiglas containers and their dimensions.

Three Plexiglas containers were constructed with dimensions 40 cm (length) × 40 cm (width) × 2 cm (height) and 3 mm thickness (Figure 4).

**Figure 5:**
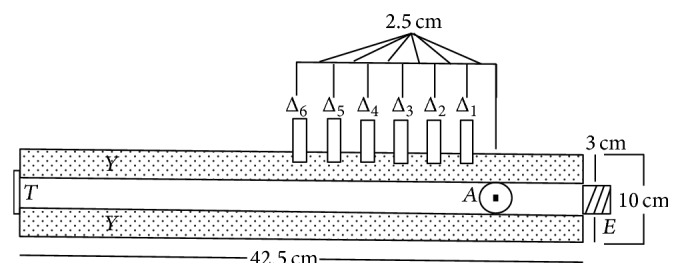
Internal detail of the cylindrical waveguide.

For the construction of the phantom the transmission line theory was used, based on the measurements of the phantom's dielectric properties. Basically, the problem is the measurement of variables *α* and *β* which constitute the real and the imaginary part of the propagation constant (*γ*). For this purpose, the coaxial line method was used [15, 16]. The coaxial line had 42.5 cm length and 10 cm diameter and its internal details are depicted in Figure 5, where (*E*) is the signal input, (Δ*i*) with *i* = 1 to 6 is the sampling, (*Y*) is the dielectric material, (*T*) is the termination of the line, and (*A*) is the reference measurement.

The transmission line theory gives

(18) $$\begin{array}{c} k=\beta -j\alpha , \end{array}$$

where *k* is the complex wavenumber $k=j\omega \sqrt{\mu_{0\left(\varepsilon_{0}\varepsilon_{r}-j\left(\sigma /\omega \varepsilon_{0}\right)\right)}}$.

So

(19) εr=β2+α2k02,σ=ωε02βαk02.

It applies

(20) β=ΔΦΔz(rad/m),

(21) α=ln⁡V1/V2Δz(Np/m).

Form (19)-(20) it results in

(22) εr=ln⁡V1/V22+ΔΦ/Δz2k02,σ=ωε02ΔΦ/Δzln⁡V1/V2/Δzk02,

where |*V*_1_|, |*V*_2_| are calculated by the amplitude of the waveform in the oscillograph that receives signal from the reference *A* and the sampling Δ*i*. The signal input (*E*) is powered by a generator at 27 MHz. The phase delay ΔΦ is measured based on the scanning frequency of the oscillograph. A computation program was developed for the *ε*_*r*_ and *σ* calculation directly from |*V*_1_|, |*V*_2_|, and ΔΦ.

Several trials have been performed for the phantom preparation based on known formulas [15] and numerous measurements of the dielectric properties have been realized using the coaxial line. Finally a soft tissue phantom was developed with *ε* = 111.4 and *σ* = 0.619 Si/m. The dielectric properties given by literature are *ε* = 113 and *σ* = 0.625 Si/m; thus our divergence is 1.4% and 0.16% for the permittivity and the conductivity, respectively. The formula for the phantom is given in Table 1.

The aluminum controls the permittivity; the NaCl controls the conductivity while the flour is mostly used for the mixture coagulation. The phantom presented two disadvantages:

- It was not solid and thus as a semifluid can be used only in horizontal applications.
- It contains flour which leads to sepsis after 3-4 days.

The three Plexiglas containers were filled with the phantom material. In order to be able to have temperature measurements from specific points, a membrane of polyethylene was placed on the surface of the material and graded axes *X*, *Y* were designed. The temperature measurements were realized with *K* type thermocouples which do not present any interference with electromagnetic fields. The air temperature was recorded and then the material temperature after 2 sec relaxation time, as the temperature difference, was finally monitored.

## 5. Results

### 5.1. Numerical Results

**Figure 6:**
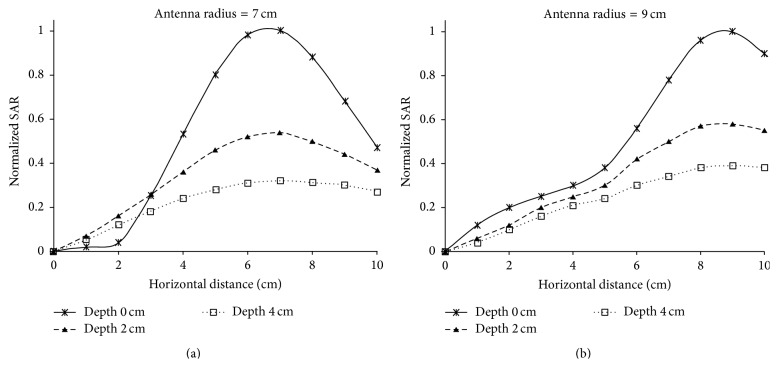
Normalized values for SAR in relation to the horizontal distance from the antenna center for three different depths and two different antennas.

**Figure 7:**
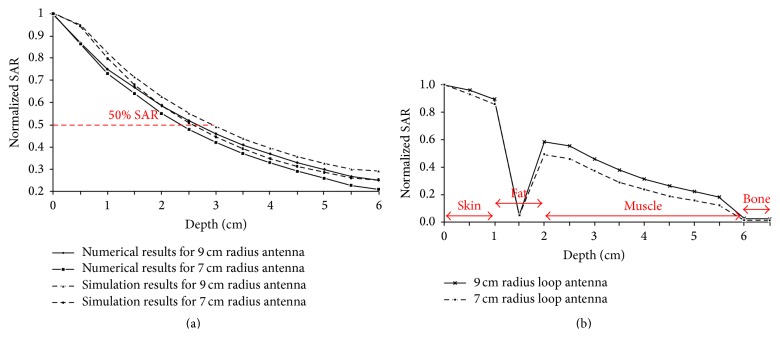
(a) Numerical and simulation results for the normalized SAR values in relation to the depth for the trace of two antennas with radii 7 cm and 9 cm placed over a dielectric semi-infinite space and a cube, respectively. (b) Simulation results for the normalized SAR values in relation to the depth for the trace of the two antennas placed over a cylinder modeled with skin, fat, muscle, and bone.

**Figure 8:**
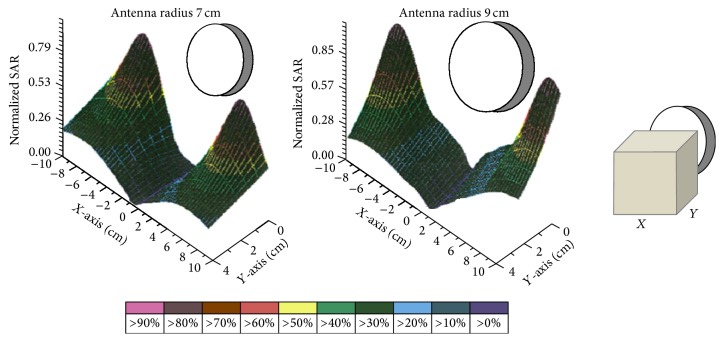
SAR distribution in relation to depth for the central level of the antennas with *R* = 7 cm and *R* = 9 cm.

Following that, numerical code executions have been realized and the results for the SAR distribution are presented for two different current loops with radii 7 cm and 9 cm. For the theoretical analysis the circular current source has been considered infinitely thin as at 27 MHz the free space wavelength is 11.1 m and thus much larger than the antenna's thickness. The dielectric properties, used for the computations, were *ε*_*r*_ = 113 and *σ* = 0.62 Si/m representing soft tissues [13]. In Figure 6 normalized values for SAR in relation to the horizontal distance from the antenna center along *OX* semiaxis are depicted. The results are given for three different depths, 0 cm, 2 cm, and 4 cm, when the radius of the antenna is 7 cm and 9 cm and is placed 1 cm above the dielectric. In Figure 7(a) the penetration of the radiation in relation to the depth is depicted for the field created by the loop antennas with radii 7 and 9 cm. Finally, in Figure 8 SAR distribution in relation to depth, for the central level (*XY*) of the antenna, is depicted, when the antenna radii are 7 cm and 9 cm.

By observing Figure 7(a) it can be concluded that the penetration depth for 50% SAR is ~2.5 cm for the two antennas, a fact that limits the application of hyperthermia to depths ranging from 2 to 3.5 cm. However, the 9 cm radius antenna gives a better penetration depth.

In Figure 6 it is shown that the SAR's maximum value is observed for the circumference of every loop, while the minimum value is observed at the center, as it was expected. This can also be concluded by observing Figure 8 where the shape of SAR distribution, with maximum values at the circumference and minimum at the center, is maintained during the penetration of the radiation. Consequently, hyperthermia using current loops is efficient in the case where the peripheral heating of spherical tumor formation located at 2–3.5 cm depth is required.

### 5.2. Simulation Results

The loop antennas with 7 cm and 9 cm radius placed 1 cm over the soft tissue cube and the cylinder were simulated with HFSS [17] at 27 MHz. In Figure 7(a) the normalized simulation results for the SAR values for the antennas in relation to the depth in the soft tissue cube are presented along with the numerical results. The penetration depth of the 9 cm radius antenna is ~3 cm, while that of the 7 cm radius antenna is ~2.5 cm.

The semi-infinite space for the theoretical analysis and the cube for the simulations have the same dielectric properties. The theoretical and simulation results present small differences, as the theoretical analysis referred to a semi-infinite dielectric.

**Figure 9:**
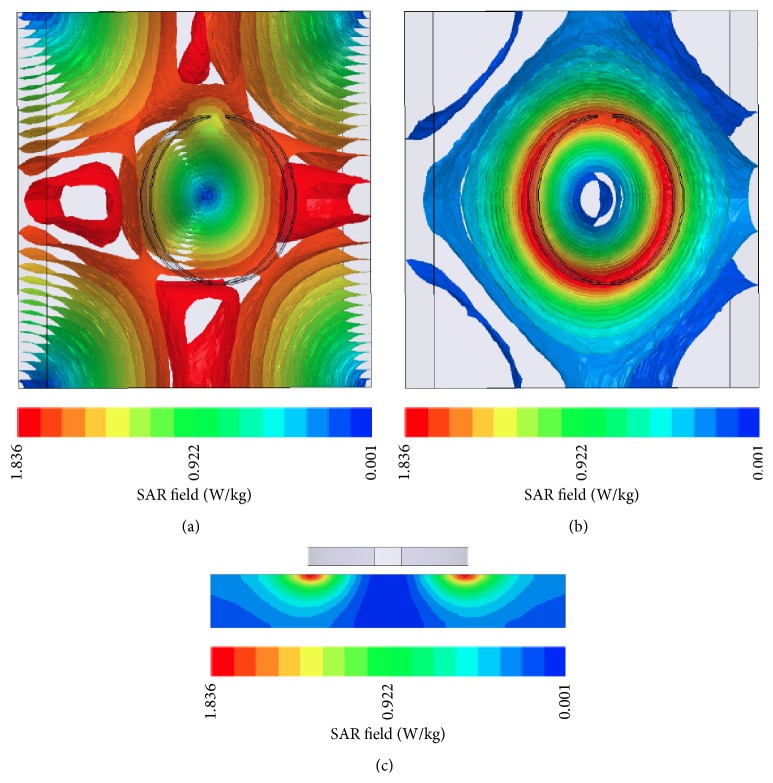
(a) Electric field and (b) SAR field inside the soft tissue cube when radiated by the 9 cm radius loop antenna.

In Figure 9 the electric field and the SAR distribution are presented for the 9 cm radius antenna. As it was expected and as the theoretical analysis showed, maximum SAR values occur at the circumference and the minimum values are at the center.

**Figure 10:**
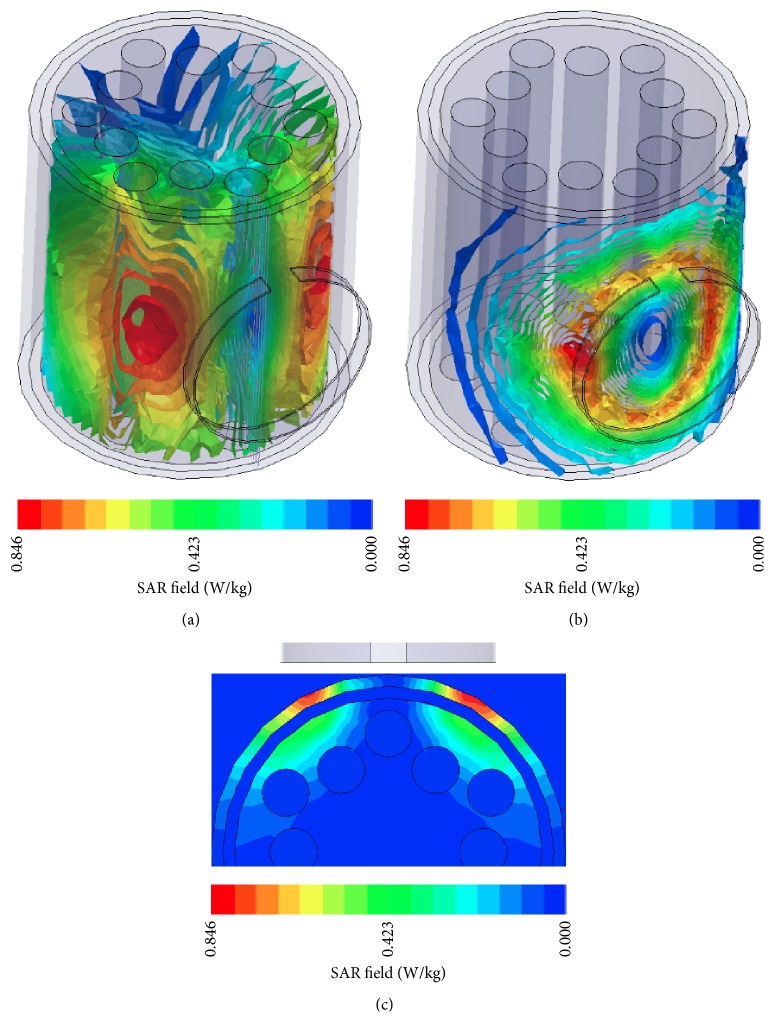
(a) Electric field and (b) SAR field inside the cylinder modeled with different body tissues when radiated by the 9 cm radius loop antenna.

The normalized SAR results in relation to the depth for the antenna-cylinder simulation setup are shown in Figure 7(b), while the electric field and the SAR distribution in the cylinder are shown in Figure 10. The penetration depth for 50% SAR is ~3 cm for the 9 cm radius antenna and ~2 cm for the 7 cm radius antenna. The layer of fat that covers the muscle tissue presents almost zero SAR. However, for the 9 cm radius antenna the results are better than the cubic simpler model.

The simulations prove that hyperthermia using the current loops for radiation is achieved at a depth of 2–4 cm.

### 5.3. Experimental Results

Due to the circular geometry of the loop, a circular symmetry for the imposed field was assumed. Measurements were realized in a quadrant and they are indicative for all *X*-*Y* plane. Along the axis of radiofrequency propagation inside the dielectric, temperature measurements were performed on three levels: on the material surface of each container.

Following, SAR values were calculated using the formula SAR = 4186 *c*Δ*T*/*t*, where *c* is the specific heat with *c* = 0.86 kcal/kg°C and *t* is the time dependence.

**Figure 11:**
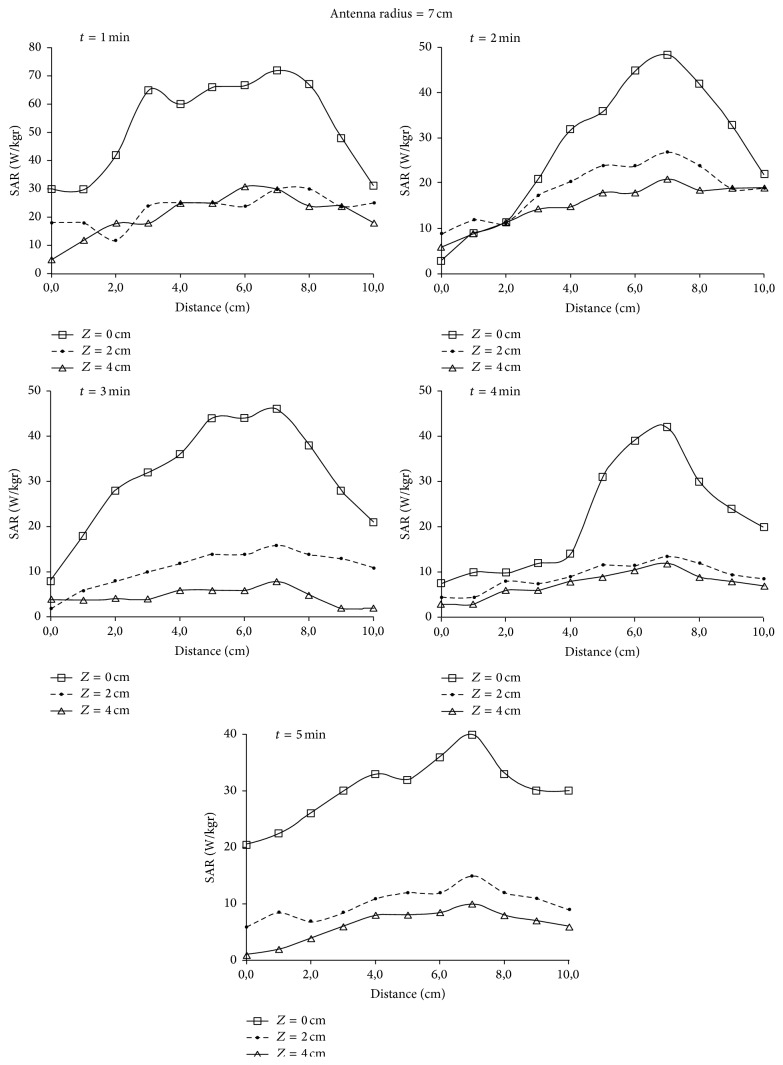
SAR distribution for depths of 0, 2, and 4 cm, for radiation times 1, 2, 3, 4, and 5 min when the antenna radius is 7 cm.

**Figure 12:**
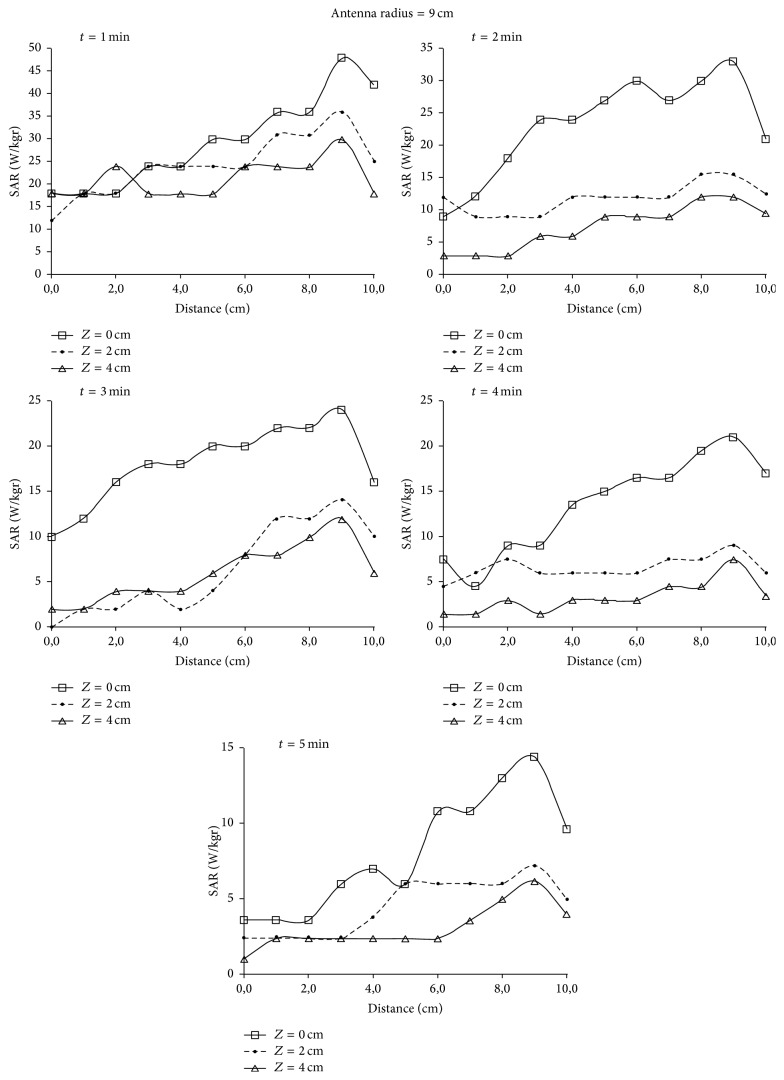
SAR distribution for depths of 0, 2, and 4 cm, for radiation times 1, 2, 3, 4, and 5 min when the antenna radius is 9 cm.

The two antennas used for the radiation had radii of 7 and 9 cm, respectively, and they were placed at 1 cm distance over the phantom material. The results are depicted in Figures 11-12 for radiation times 1, 2, 3, 4, and 5 min, depths 0, 2, and 4 cm, and distances from the center of the circular loop 0–10 cm with a step of 1 cm. In Figure 10 the SAR distribution in relation to the penetration depth in the phantom is depicted.

## 6. Discussion and Conclusions

The proposed inductive loop was designed as a potential tool for hyperthermia treatment utilizing external antennas for electromagnetic field energy application. More specifically, in this paper, the performance of a 27 MHz inductive loop antenna was studied theoretically as well as phantom experiments.

The theoretical results showed that SAR's maximum value was observed for the circumference of every loop, while the minimum value is observed at the center, as it was expected. In addition the shape of SAR distribution, with maximum values at the circumference and minimum at the center, was maintained during the penetration of the radiation. Consequently hyperthermia using current loops is efficient in the case where the peripheral heating of spherical tumor formation is required.

For the phantom, a clear identification between the experimental values of the SAR measurements and the theoretical values was observed. Small deviations are justified by the temperature dissemination and the temperature exchanges with the environment.

A satisfactorily SAR curve was observed with smoothing and flattening of SAR values inside the loop, while a significant increase in SAR values was shown for the phantom areas which were radiated by the antenna's periphery. In addition a decrease of SAR values was observed as the radiation time was increased. This is justified by the temperature dissemination inside the dielectric material and by the energy exchanges between the phantom and the environment during the radiation. Consequently, the temperature measurements must be realized for radiation time <2 min.

The potential clinical application of the suggested hyperthermia loop-applicator would be the heating of abdominal cavity which might be achieved with the temperature elevation of liquid element in generalized-defused peritoneal carcinomatosis in conjunction with chemotherapy [18, 19]. Circular and loop antennas have been used by other research groups at a variety of operation frequencies showing comparable results to those presented in this paper.

Hwang et al. [10] used a waveguide circular antenna with fan at operating frequency 5.8 GHz, for concentrating the radiated EM to the adipose tissue under skin. The observed difference in temperature was about 3°C.

Ishikawa et al. [11] used a loop antenna, operating at 430 MHz, set in water with temperature kept at 25°C. The muscle with initial temperature of 37°C was set above them and the distance between muscle and antenna was 30 mm. The circular loop antenna could heat muscle tissue above 42°C and the heated region was observed at 14 mm inside from bottom surface of muscle.

Yabuhara et al. [12] proposed a system where the human head was placed in the gap of two reentrant cylinders inside a cylindrical cavity and it was heated with electromagnetic fields excited by a loop antenna of 70 mm diameter and operating frequency that could be changed from 50 to 200 MHz. Computer simulation and experimental results with an agar phantom showed that the system could be applicable to hyperthermia treatment of deep and surface brain tumors.

Gerbaux et al. [9] used a resonant loop antenna of radius *r* = 101.5 mm at 434 MHz placed 50 mm above a human body model to calculate the specific absorption rate (SAR). Two local peaks of SAR of 0.1 W/kg per watt antenna input power were observed, one below the antenna port and one below the opposite side of the loop.

In the loop antenna setup presented in this paper, in the three-dimensional SAR distribution in relation to the penetration depth of the radiation for the central level of both radiators, significantly high values were observed while the penetration depth (50% SAR) was 2.5 cm. A way to avoid the dissimilarity and limitation of the proposed setup is the use of a folded loop antenna as a radiator in order to destroy the uniformity of the magnetic field and the induced uniformity in the electric field [20].

## Figures and Tables

**Table 1:** Dielectric properties of body tissues used for the cylindrical structure.

	Electrical	Loss tangent	Conductivity
	permittivity	(tan⁡*δ*)	(Si/m)
Skin	114.74	2.48	0.427
Fat	8.468	2.587	0.033
Muscle	95.95	4.538	0.654
Bone	42.01	2.248	0.382

**Table 2:** The formula for the phantom.

Element	NaCl	Al	flour	H_2_O
Content	0.27%	3.5%	15.2%	81%
